# Low-dose zoledronate for the treatment of bone metastasis secondary to prostate cancer

**DOI:** 10.1186/s12935-019-0745-x

**Published:** 2019-02-08

**Authors:** Elie Akoury, Pouyan Ahangar, Antone Nour, Jacques Lapointe, Karl-Philippe Guérard, Lisbet Haglund, Derek H. Rosenzweig, Michael H. Weber

**Affiliations:** 1 Department of Surgery, Division of Orthopaedics, McGill University and The Research Institute of the McGill University Health Centre, Injury Repair Recovery Program, Montreal, QC Canada; 2 Department of Surgery, Division of Urology, McGill University and The Research Institute of the McGill University Health Centre, Cancer Research Program, Montreal, QC Canada

**Keywords:** Bone metastases secondary to prostate, Zoledronate, Direct in vitro treatment, Low doses, Cellular assays

## Abstract

**Background:**

Bisphosphonates (BPs) including zoledronate (zol) have become standard care for bone metastases as they effectively inhibit tumor-induced osteolysis and associated pain. Several studies have also suggested that zol has direct anti-tumor activity. Systemic administration at high doses is the current approach to deliver zol, yet it has been associated with debilitating side effects. Local therapeutic delivery offers the ability to administer much lower total dosage, while at the same time maintaining sustained high-local drug concentration directly at the target treatment site. Here, we aimed to assess effects of lower doses of zol on bone metastases over a longer time.

**Methods:**

Prostate cancer cell line LAPC4 and prostate-induced bone metastasis cells were treated with zol at 1, 3 and 10 µM for 7 days. Following treatment, cell proliferation was assessed using Almarblue^®^, Vybrant MTT^®^, and Live/Dead^®^ viability/cytotoxicity assays. Additionally, cell migration and invasion were carried out using Falcon™ cell culture inserts and Cultrex^®^ 3D spheroid cell invasion assays respectively.

**Results:**

We show that treatment with 3–10 µM zol over 7-days significantly decreased cell proliferation in both the prostate cancer cell line LAPC4 and cells from spine metastases secondary to prostate cancer. Using the same low-dose and longer time course for treatment, we demonstrate that 10 µM zol also significantly inhibits tumor cell migration and 3D-cell growth/invasion.

**Conclusions:**

This project harnesses the potential of using zol at low doses for longer treatment periods, which may be a viable treatment modality when coupled with biomaterials or biodevices for local delivery.

**Electronic supplementary material:**

The online version of this article (10.1186/s12935-019-0745-x) contains supplementary material, which is available to authorized users.

## Background

Bone is the most common site for metastasis, often arising from solid tumors affecting the breast, lung, prostate and other sites [1–4]. These bone tumors are a major cause of morbidity and can be associated with deleterious effects known as skeletal related events (SREs). SREs encompass severe pain, impaired mobility, pathologic fractures and spinal cord compression, all of which may profoundly impair a patient’s quality of life. With advances in medical, radiological and surgical treatment options, spine metastasis patients are living longer, and the focus is now shifting to improving the outcomes and quality of life. Novel treatment development therefore aims to slow the recurrence of tumors, repair damaged tissues, alleviate symptoms and pain, avoid therapeutic side effects and improve the patient’s well-being [5].

A major treatment strategy for many spine metastases is surgical excision, which is often extensive to ensure removal of all malignant tissue and to prevent tumor recurrence. The resulting defect is often large and will not heal spontaneously. Autologous bone grafting and stabilizing is commonly applied, yet donor site morbidity and amount of graft materials is often a concern. Other available therapeutic modalities used in combination with surgery include radiotherapy, chemotherapy, hormone therapy and the use of BPs [6].

BPs were classically viewed as a first line treatment for patients with osteoporosis [7], Paget’s disease [8] and hypercalcemic disorders [9]. Zoledronate or zoledronic acid (zol), is a third-generation nitrogen-containing BP, and the most potent BP described so far. Zol has been proven to be clinically beneficial mostly in reducing the incidence of SREs not only in patients with prostate-induced bone metastasis [10], but also in those with bone metastases arising from other primary cancers such breast and myeloma [11–13]. Additionally, zol treatment has been shown to prevent osteoclast-mediated bone resorption [14] and reduce the growth of primary bone tumors as well as bone metastases of prostate, lung, breast, and other solid tumors [15–17].

The mechanism of action underlying the anti-tumor effect of zol is not fully understood. However, investigations have demonstrated the role of zol in the induction of tumor cell apoptosis [18–20], modulation of the immune system [21, 22], inhibition of tumor invasion [23, 24], decrease of tumor proliferation [25–27] and reduction of tumor angiogenesis [28–30]. The current standard for delivering zol to patients with bone metastasis is via intravenous infusion (for 15 min), and the recommended treatment dose is 4 mg every 3–4 weeks [30]. However, such prolonged and high systemic administration of zol can cause multiple adverse effects, ranging from flu-like symptoms to osteonecrosis of the jaw and renal toxicity [31].

To overcome the debilitating side effects of systemic zol administration, local delivery is an attractive alternative option. Many recent studies have explored the potential of local BP delivery, including zol, in implants and animal models [32–35]. Additionally, our group has demonstrated that direct local zol delivery at the site of bone tumor decreases tumor proliferation, increases tumor apoptosis and significantly blocks tumor-induced osteolysis to a greater degree than systemic therapy [36]. In the present study, we use a series of functional assays to test the hypothesis that sustained low-dose zol treatment will effectively inhibit tumor cell growth, migration and invasion. The prostate cancer cell line LAPC4 and primary cells from spine metastases secondary to prostate cancer were used in in vitro experiments. We demonstrate that ≤ 10 µM zol treatment significantly decreases cell proliferation, cell migration and 3D spheroid matrix invasion of both LAPC4 and prostate-induced bone metastasis cells. Results from this study highlight the anti-tumor effect of prolonged low-dose zol treatment of prostate-related bone metastases in clinically relevant cell populations.

## Methods

### Cell lines and prostate-induced bone metastasis cells

Human prostate cancer immortalized cell line LAPC4 was obtained from Dr. Robert Reither’s laboratory, UCLA. Collection of patient samples was approved by the institutional review board of McGill University (IRB# BMD-10-118). Tumor biopsies were resected from a patient with bone metastasis secondary to prostate cancer. Tissue samples were washed with sterile PBS1x (USA, Sigma—cat D5652) and cut into 2 mm × 2 mm sections for processing. Samples were incubated at 37 °C overnight in a 1.5 mg/ml collagenase (USA, Thermofisher, Gibco—17101-015) bath in standard RPMI 1640 growth medium, 10% Fetal bovine serum (FBS) (USA, Gibco, Thermofisher—cat 12483-020), and digested cells were isolated the next day following straining in a 70 µM cell strainer, and then pelleting in a centrifuge at 1000×*g* for 5 min. Isolated cells consisting of a mixed population of bone metastasis cells and bone/stromal cells were cultured in an RPMI cell culture medium (USA, Gibco, Thermofisher—cat 11835-030) supplemented with 10% FBS, 1% penicillin/streptomycin (PS) (USA, Gibco, Thermofisher—cat 15070-063), 1% glutamax (USA, Gibco, Thermofisher—cat 35050-061), 1% fungizone (USA, Gibco, Thermofisher—15290-018) at 37 °C in a humidified atmosphere of 5% carbon dioxide (CO_2_).

### Proliferation assay

Proliferation was evaluated using both Alamarblue^®^ kit (USA, Thermofisher—cat DAL1025) and Vybrant^®^ MTT cell proliferation kit (USA, Thermofisher—cat V13154) according to the protocols provided by the manufacturers. Briefly, LAPC4 and prostate-induced bone metastasis cells were seeded at a density of 5000 cells/well in 96 well plates (USA, Costar, FisherScientific—cat 3882) coated with poly-l-lysine (USA, Sigma—cat P4707-50ML) and were grown in standard conditions (RPMI, 10% FBS, 1% PS) for 24 h. The next day, cells were treated with vehicle (PBS1x) or zol (USA, Sigma—cat SML0223-50MG) in low-serum conditions (1% FBS) for 7 days. The media was replaced (with either drug or vehicle) on day 4 for each experiment. For alamarblue^®^ assay, almarBlue dye was added to media at 1:10 dilution on day 7 and cells were incubated at 37 °C for 4 h. For Vybrant^®^ MTT cell proliferation assay, the cells were labelled with MTT at 1:10 dilution on day 7 and incubated for 4 h at 37 °C. Then, 75 µl of media containing MTT was removed from each well before adding 50 µl of DMSO (USA, Sigma– cat D2438) for each well and incubating cells for 10 min at 37 °C. After incubation, fluorescence of alamarblue (Excitation—540 nm, Emission 585) or the absorbance of MTT (540 nm) was analyzed using the Infinite Tecan M200 Pro microplate reader (Tecan Trading AG, Männedorf, Switzerland).

### Live/Dead^®^ viability/cytotoxicity assay

Live/Dead^®^ viability/cytotoxicity assay was performed as previously described [37, 38]. Briefly, the cells that were previously assayed for alamarblue^®^ in 96 well plate, were washed with PBS1x before 100 µl of live/dead mix (2 µM calcein AM and 4 µM ethidium homodimer-1 (EthD-1) diluted in 1 ml PBS1x) (USA, Themofisher—cat L3224) was added to each well. The cells were incubated at room temperature for 20–40 min and imaged using an inverted fluorescence microscope (USA, Olympus, IX71) at 4× magnification and cells were counted. Live cells were labelled green (calcein AM) and dead cells were stained red (EthD-1).

### Migration assay

To test migration, LAPC4 were seeded at a density of 20,000 cells/well in the upper compartment of Falcon™ cell culture inserts (8 µm pore size; Canada, Falcon—cat 353097) coated with poly-l-lysine. The next day, LAPC4 were treated with vehicle or zol at different concentrations in low-serum conditions (1% FBS) in the upper compartment. Cell migration was triggered for 7 days with the use of vehicle or drug-containing RPMI supplemented with 2% FBS media as a chemoattractant in the lower compartment. After migration through the filter, the cells of both compartments were assayed for alamarblue to check for cell proliferation. The cells of the upper compartment of the insert were then removed with cotton swabs, and those on the lower compartment were fixed with 4% paraformaldehyde (USA, Thermofisher—cat 28908), counterstained with DAPI (USA, Sigma—cat F6057-20ML) and imaged using an inverted microscope (USA, Olympus, IX71) before counting. Each treatment was done in triplicate and the migrated cell number was quantified by counting at least five random fields.

### Spheroid growth and invasion assay

Spheroid growth of LAPC4 or prostate-induced bone metastasis cells was assessed using Cultrex^®^ 3D Spheroid Cell Invasion Assay (USA, Thermofisher—cat 3500-096-K) according to the manufacturer’s instructions. Briefly, cells were seeded at a density of 5000 cells/well in a low adherent 96 well plate in standard conditions (RPMI, 10% FBS) with spheroid formation extracellular matrix buffer and incubated for 3 days until spheroids are formed. Spheroid growth in the presence or absence of zol was monitored daily over a course of 14 days using an inverted microscope (USA, Zeiss, Axiovert 40 C) at 10× magnification. The spheroid area was measured using image J software (USA, NIH, version 1.51J8). In parallel, the metabolic activity of the cells in the matrix was assessed using alamarblue^®^ assay. For invasion, spheroids were embedded in an invasion matrix before treatment with vehicle or zol. Then the area of the cells that invaded the matrix was assessed as described above. The area of either drug-treated cells or vehicle-treated cells of each day was normalized to day 0 and then all normalized conditions were normalized to vehicle (PBS1x). Also, spheroids in either growth or invasion assay were assayed with alamarblue to check for cell proliferation at day 14.

### Statistics

Data from each experiment was transferred to Excel datasheet (Microsoft Office 2016). Statistical analyses were performed using R v.3.4.1 (The R Core Team, 2016) and R studio Software (USA, version 1.1.453). All data are expressed as the mean ± SD. Comparisons between groups were made by one-way or two-way ANOVA and Tukey post hoc tests at a 95% confidence level. When heteroscedasticity was present White’s adjust was performed. p values < 0.05 were considered as statistically significant.

## Results

### Zoledronate affects the proliferation of prostate cancer cell line LAPC4

**Fig. 1:**
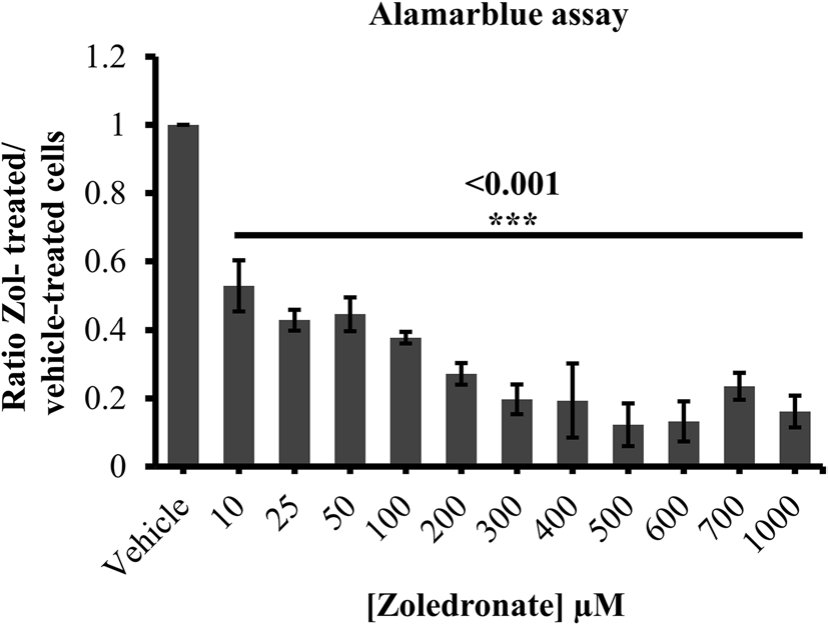
Cell proliferation using alamarblue^®^ assay of LAPC4 cells treated with vehicle (PBS1x) or zol 10 to 1000 µM for 7 days in 1% serum conditions. The histogram represents the ratio of drug-treated cells divided by vehicle-treated cells (PBS1x). Results are the mean ± SD of three independent experiments, p < 0.05

**Fig. 2:**
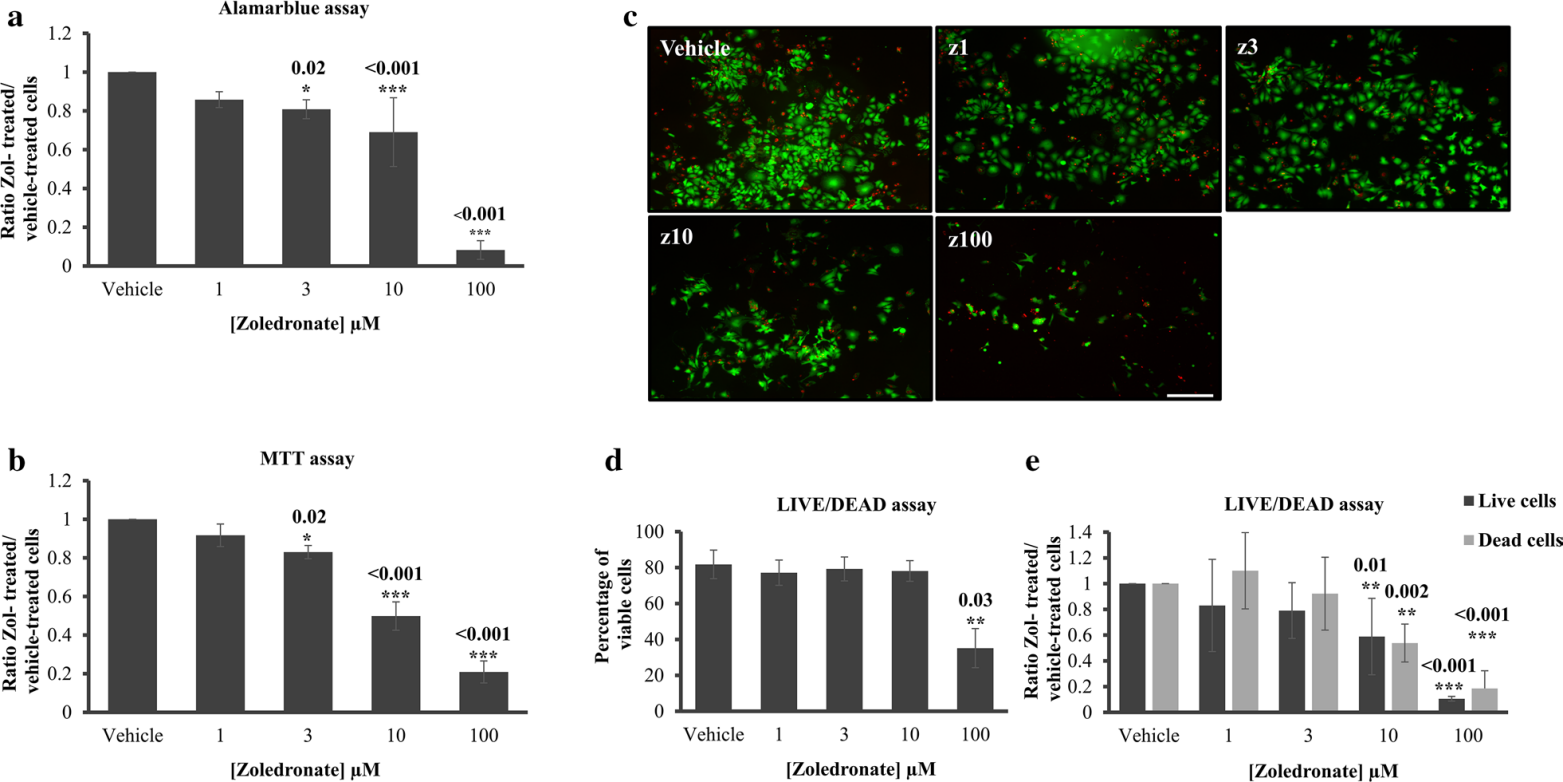
Cell proliferation using alamarblue^®^ assay (**a**) and Vybrant MTT^®^ assay (**b**) of LAPC4 cells treated with vehicle (PBS1x) or zoledronate 1 µM, 3 µM, 10 µM and 100 µM for 7 days in 1% serum conditions. The histograms in (**a**) and (**b**) represent the ratio of drug-treated cells divided by vehicle-treated cells (PBS1x) in three independent experiments for all variables except for 100 µM that was done as a single experiment in triplicate. **c** Representative photos of Live/Dead assay carried out on LAPC4 following vehicle or zol treatment at different concentrations. Live cells are in green and dead cells are in red. **d** Percentage of viable cells [number of live cells/(number of live cells + number of dead cells) * 100] and **e** ratio of live cells or dead cells in vehicle or zol-treated conditions, Results are mean ± SD, p < 0.05. Scale bar 250 µm

Short-term and systemic delivery of high-dose zol has the potential to cause debilitating systemic side effects [31]. Precision medicine is therefore exploring the potential of local drug delivery using lower overall doses for longer treatment periods to achieve adequate anti-cancer activity at the site of tumors while preventing unwanted systemic side effects. At the same time, sustained local drug release leads to high local concentrations which will presumably be more effective at the target site. To establish an effective low-dose range for zol treatment over a longer time course, a 10–1000 µM range of zol was applied to the prostate cancer cell line LAPC4 in 2D culture (Fig. 1). Using alamarblue^®^ assay, we observed a statistically significant, dose dependent decrease in LAPC4 cell proliferation starting at 10 µm (47.2% ± 7.4% to 84 ± 4.6%, p value < 0.001) in zol-treated cells as compared to vehicle-treated cells. Additionally, we determined the EC_50_ dose for zol on LAPC4 cells to be 3.8 µM. LAPC4 cells were treated with concentrations of zol either lower, at, or higher than the EC50 (1, 3, 10 and 100 µM) for 7 days in low serum conditions (1% FBS) (Fig. 2a). In a dose dependent manner, zol significantly decreased LAPC4 cell proliferation at 3 µM (19% ± 4.8%, p value = 0.02),10 µM (31% ± 17.7%, p value < 0.001) and 100 µM (91.8% ± 4.8%, p value < 0.001), but not at 1 µM. The Vybrant^®^ MTT cell proliferation assay also showed similar significant decreases at 3 µM (17% ± 3.4%, p value = 0.02), 10 µM (50% ± 7.36, p value = 0.001) and 100 µM (79.2% ± 5.6%, p value < 0.001)—zol treatment when compared to untreated cells (Fig. 2b). We also tested effects of 1, 3, 10 and 100 µM zol treatment of LAPC4 cells over 14 days, with the results being quite similar to 7-day treatment with the exception of slightly further decrease in metabolic activity with 100 µM zol treatment (Additional file 1: Figure S1). To further validate these observations, Live/Dead^®^ assay was performed and showed that treatment with 1, 3 and 10 µM zol did not affect the percent-viable cells, yet the total number of cells was significantly decreased after 10 µM zol treatment (live cells 41.1% ± 29.7%, p value = 0.01, dead cells 46.1% ± 14.7%, p value = 0.002) (Fig. 2c–e). However, 7-day treatment with 100 µM decreased significantly but not drastically the percent-viable cells (59.1% ± 16.8% p value = 0.03) and the total number of live and dead cells (live cells 89.5% ± 1.82%, p value < 0.001, dead cells 81.4% ± 13.7.7%, p value < 0.001) (Fig. 2 c–e). After 14-day treatment, viability was similar to 7-day treatment, with the exception that 100 µM caused almost complete cell death (Additional file 1: Figure S1). Taken together, these data suggest that low-dose zol treatment (1–10 µM) over 7–14 days blocks LAPC4 cell proliferation.

### Zoledronate affects the proliferation of prostate-induced bone metastasis cells

**Fig. 3:**
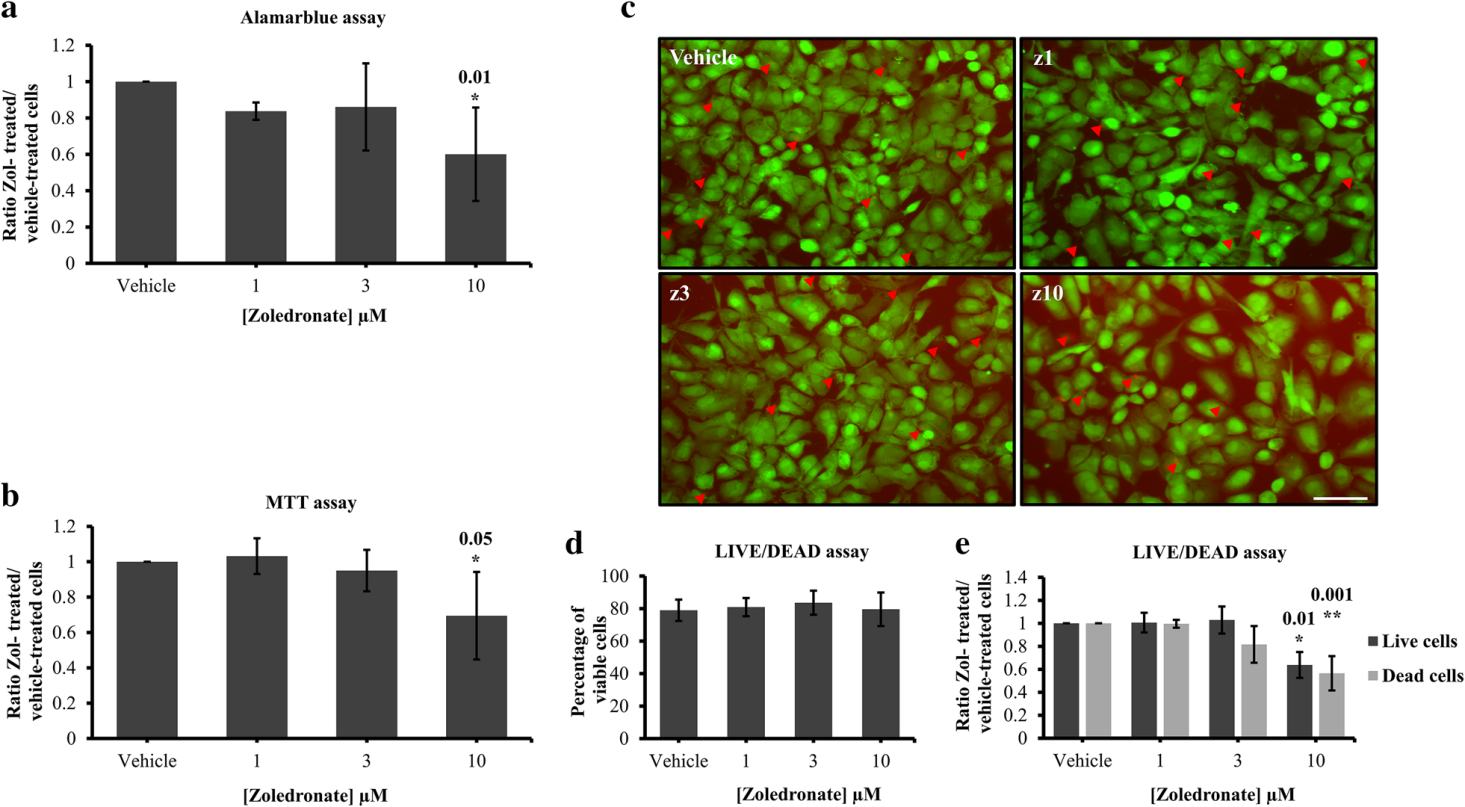
Cell proliferation using alamarblue^®^ assay (**a**) and Vybrant MTT^®^ assay (**b**) of prostate-induced bone metastasis cells treated with vehicle (PBS1x) or zol 1 µM, 3 µM and 10 µM for 7 days in 1% serum conditions. The histograms in (**a**) and (**b**) represent the ratio of drug-treated cells divided by vehicle-treated cells (PBS1x). **c** representative photos of Live/Dead assay carried out on LAPC4 following vehicle or zol treatment at different concentrations. Live cells are in green and dead cells are designated in red arrow heads. **d** Percentage of viable cells [number of live cells/(number of live cells + number of dead cells) * 100] and **e** ratio of live cells or dead cells in vehicle or zol-treated conditions, Results are mean ± SD of three independent experiments, p < 0.05. Scale bar 100 µm

To test the effects of zol treatment in a more clinically relevant manner, we applied the same range of low-dose zol (1, 3 and 10 µM over 7 days) on isolated spine metastases cells secondary to prostate cancer. Treatment of these cells with 10 µM zol followed by alamarblue^®^ assay significantly reduced proliferation compared to vehicle control (40% ± 25.7%, p value = 0.01) (Fig. 3a). MTT assay also showed a similar significant reduction in prostate-induced bone metastasis cell proliferation with 10 µM zol (30.6% ± 24.7%, p value = 0.05) (Fig. 3b). Using Live/Dead^®^ assay, we confirmed that the percentage of viability of these bone metastasis cells remained the same (80%) in all conditions. Similar to treatment of LAPC4 prostate cancer cells, we found also in bone-derived cancer cells secondary to prostate cancer a significant reduction in total cell numbers following zol 10 µM treatment [live cells (36.2% ± 22.5, p value = 0.01), dead cells (43.4% ± 16.8, p value = 0.001)] (Fig. 3c–e). All three cellular assays indicate that treatment with zol 10 µM slows down cell proliferation of prostate-induced bone metastasis cells.

### Zoledronate affects the migration of LAPC4 and prostate-induced bone metastasis cells

**Fig. 4:**
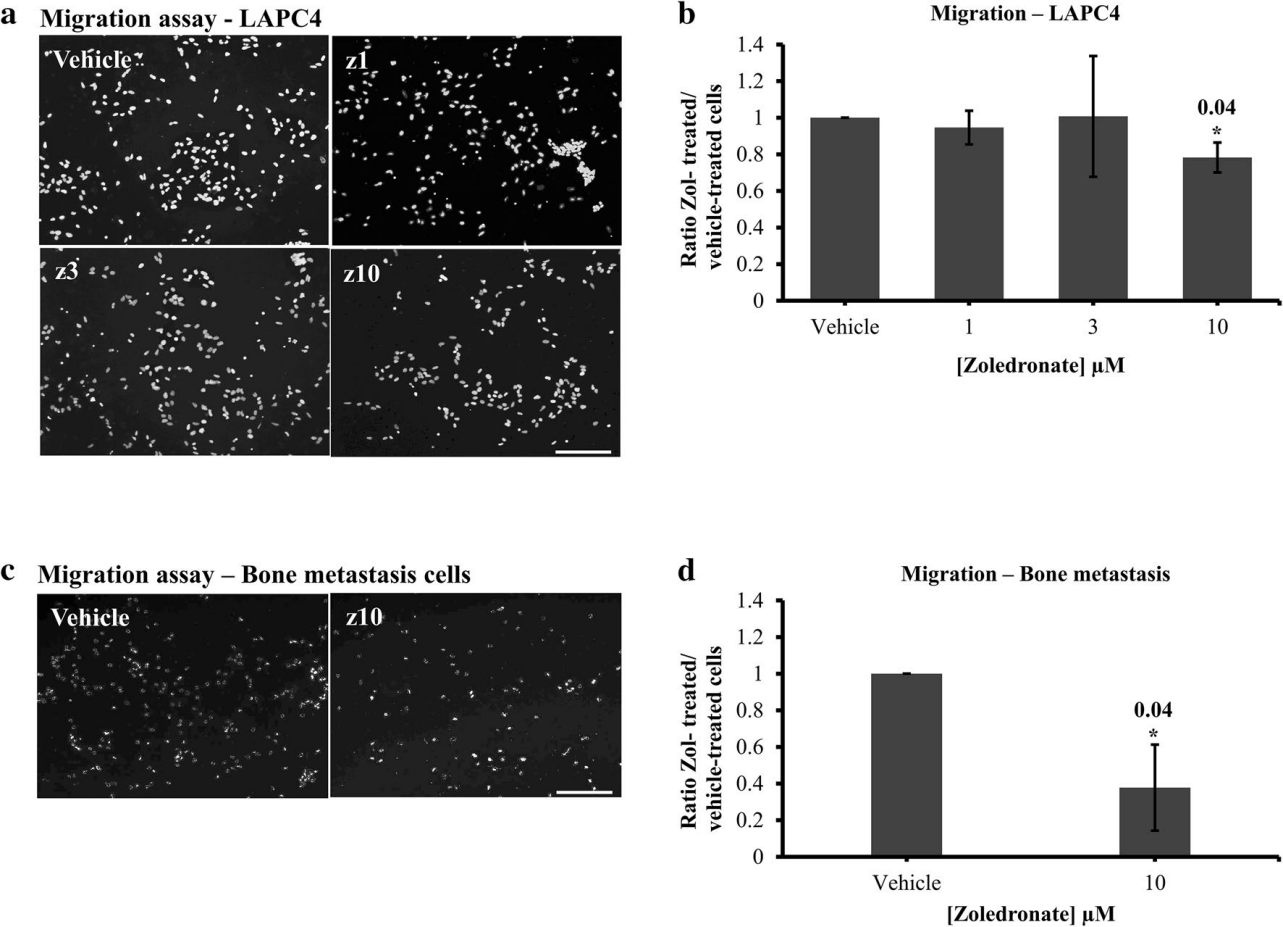
Migration (Falcon™ insert assay) of LAPC4 (**a** and **b**) and prostate-induced bone metastasis cells (**c** and **d**) treated with vehicle (PBS1x) or zol 1 µM, 3 µM and 10 µM for 7 days in 1% serum conditions. Representative images of LAPC4 (**a**) and prostate-induced bone metastasis patient cells (**c**) cells from vehicle or zol-treated conditions. The histograms represent the ratio of drug-treated cells divided by vehicle-treated cells (PBS1x) for LAPC4 (**b**) and prostate-induced bone metastasis cells (**d**). Results are the mean ± SD of three independent experiments, p < 0.05. Scale bar 100 µm

To determine whether low-dose zol treatment can disrupt tumor cell migration, trans-well Falcon™ chamber assays were performed in the same range of zol concentrations (1, 3 and 10 µM) over 7 days. Compared to vehicle control, we found that LAPC4 cell migration to the underside of the transwell was significantly decreased following treatment with 10 µM zol (22.8% ± 8.1, p value = 0.04) following 1-week treatment (Fig. 4a, b). Treatment of prostate-induced bone metastasis cells with 10 µM zol also significantly decreased migration of cells compared to control (62.3 ± 23.4%, p value = 0.04) (Fig. 4c, d). These data indicate that low-dose zol treatment over 7 days can effectively inhibit tumor cell migration. Both LAPC4 and prostate-induced bone metastasis cells treated with 10 µM showed a significant decrease in proliferation after 1-week migration (data not shown).

### Zoledronate affects spheroid growth and invasion of LAPC4 and prostate-induced bone metastasis cells

**Fig. 5:**
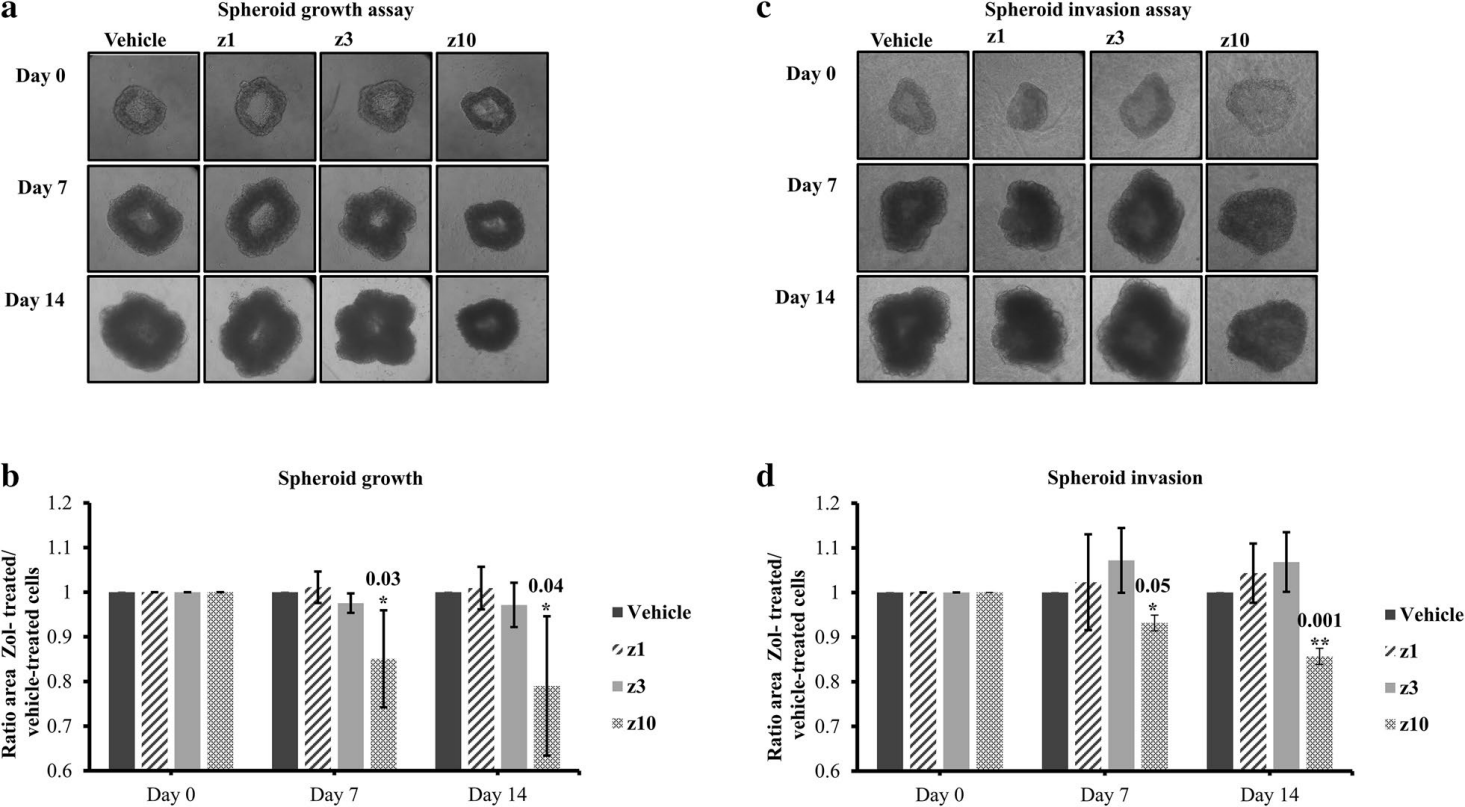
Spheroid growth (**a** and **b**) and spheroid invasion (**c** and **d**) of LAPC4 cells treated with vehicle (PBS1x) or Zol 1 µM, 3 µM and 10 µM for 7 and 14 days in 1% serum conditions. Representative brightfield images of LAPC4 spheroids growth (**a**) and invasion (**c**) from vehicle or zol-treated conditions. Histograms for growth (**b**) and invasion (**d**) showing the mean ± SD of three independent experiments, p < 0.05. Each condition (drug-treated cells or vehicle-treated cells) on each day was normalized to day 0 and then all normalized conditions were normalized to vehicle (PBS1x)

To determine whether low-dose zol treatment can disrupt tumor cell spheroid growth or invasion, a 3D-culture spheroid assay in basement membrane matrix was used. Formed LAPC4 spheroids were treated with zol at different concentrations, and the spheroid surface area was measured over 14 days (Fig. 5a, b). LAPC4 spheroids treated with 10 µM zol were significantly smaller than vehicle-treated spheroids at both 7 (15% ± 10.8%, p value = 0.03) and 14 days (21% ± 15.5%, p value = 0.04) (Fig. 5a and b). Treating spheroids with lower concentrations (1 and 3 µM) of zol did not show any significant effect from day 1 to day 14 (Fig. 5a, b). Furthermore, the spheroids treated with 10 µM zol also showed a significant decrease in proliferation (data not shown).

When LAPC4 spheroids were encapsulated in a 3D-invasion matrix and treated with zol at 1, 3 and 10 µM for 14 days, the cells as expected did not show any spindle-like protrusions which reflect the process of cell invasion [39] in either the drug-treated or untreated cells (Fig. 5c, d). Similarly to the spheroid growth area analysis, LAPC4 spheroids expansion within invasion matrix was significantly smaller when treated with 10 µM zol, as compared to controls at both 7 days (6.9% ± 1.7%, p value = 0.05) and 14 days (14.4% ± 1.8%, p value = 0.001) (Fig. 5c, d). No significant changes in the spheroid surface area was observed for the cells treated with lower concentrations of zol 1 µM or 3 µM when compared to those treated with vehicle during the entire 14-day treatment (Fig. 5c, d).

**Fig. 6:**
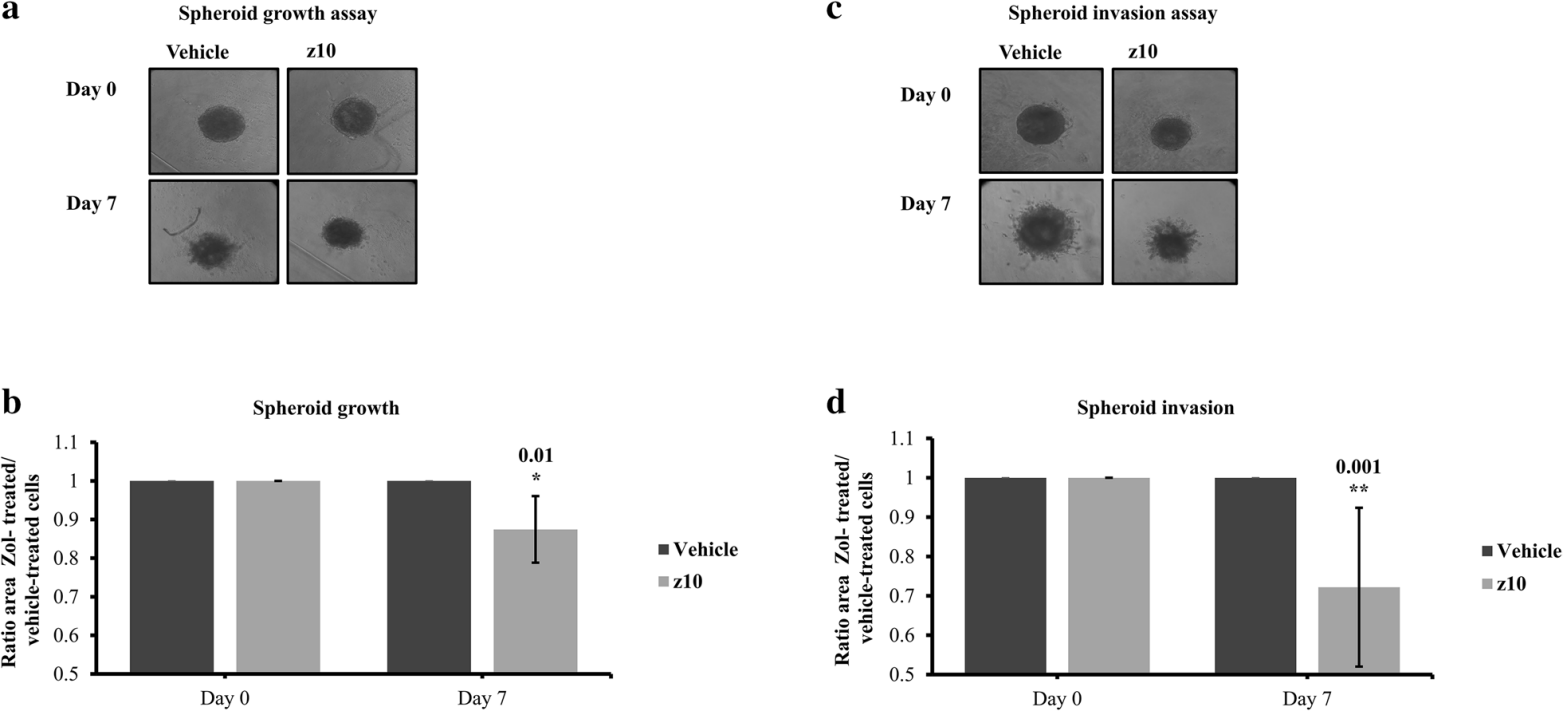
Spheroid growth (**a** and **b**) and spheroid Invasion (**c** and **d**) assays of prostate-induced bone metastasis cells treated with vehicle (PBS1x) or zol 10 µM for 7 days in 1% serum conditions. Representative brightfield images of spheroids growth (**a**) and invasion (**c**) of prostate-induced bone metastasis cells from vehicle or zol-treated conditions. Histograms for spheroids growth (**b**) and invasion (**d**) showing the mean ± SD of three independent experiments, p < 0.05. Each condition (drug-treated cells or vehicle-treated cells) on each day was normalized to day 0 and then all normalized conditions were again normalized to vehicle (PBS1x)

We also assessed spheroid growth and invasion of the prostate-induced bone metastasis cells in the same manner. Treatment of these cells with 10 µM zol over 7 days significantly reduced spheroid surface area (12.6% ± 8.6%, p value = 0.01) (Fig. 6a, b) and outgrowth into embedded matrix (27.9% ± 20.16%, p value = 0.001) (Fig. 6c, d). Interestingly, prostate-induced bone metastasis cells were able to migrate out of the spheroids and invade the surrounding matrix in untreated controls. This was however abolished in those treated with 10 µM zol. When considering spheroid and matrix invasion for both LAPC4 and prostate-induced bone metastasis cells, treatment with low-dose zol over 7 days can significantly reduce tumor growth, individual cell outgrowth, and proliferation (data not shown) while in a more physiological 3D culture.

## Discussion

Finding a curable treatment for bone metastases remains a considerable challenge. Patients presenting with bone metastases secondary to prostate are offered few approved therapeutic modalities which do not cure the disease but aim instead to alleviate the symptoms and provide a better quality of life for patients. Zol is one of few treatment options that is available. However, zol is administered systemically at high doses to achieve therapeutic effects. High systemic zol dosage has been related to severe side effects in patients with bone metastases. Indeed, several chemotherapeutics are associated with severe side effects following high systemic dosage. This has prompted a shift in research focus toward local and targeted drug delivery to maintain high drug concentrations directly at the site needed and avoid such negative side effects. In this study, we evaluated for the first time the anti-tumor properties of low-dose (1, 3 and 10 µM) zol treatment over 7 days on LAPC4 prostate cancer cell line which was previously established following harvesting from the lymph node metastasis of a male patient with hormone refractory prostate cancer [40]. Following 1-week treatment, zol significantly reduced LAPC4 cell proliferation while maintaining the ratio between live and dead cells at 10 µM and to a lesser extent at 3 µM. Interestingly, the same observation applies for 10 µM treatment of the clinically relevant prostate-induced bone metastasis cells that were treated in the same conditions. These data indicate that zol at lower concentrations (≤ 10 µM) reduce proliferation but does not kill the prostate cancer cell line or the prostate-induced bone metastasis cells in 2D culture for a time course of 7 days. Unfortunately, we did not obtain normal, healthy osteoblastic or stromal cells from the bone metastasis patient derived samples to test whether low-dose zol equally affects normal cell proliferation and viability. Therefore, future studies will determine effects of zol on normal cell homeostasis.

Since cell migration is a key-step in tumor invasion and metastasis [41], we used Falcon™ cell culture inserts to assess this cellular process following 7-day zol (1, 3 and 10 µM) treatment on LAPC4 and prostate-induced bone metastasis cells. Following analysis, we found significantly reduced cell migration in cultures treated with 10 µM zol. This could be explained by the fact that the gradient of serum (> 1%) used in the transwell chamber cell migration assay may have masked the effect of zol at lower concentration (3 µM) in LAPC4. Alternatively, one could also envision that the minimal dose of zol required to reduce LAPC4 migration is at least 10 µM. Although possibly true for LAPC4, this might not be the case for the prostate-induced bone metastasis patient cells which in fact required 10 µM to impair migration as well as proliferation.

Conventional 2D monolayer culture lacks many environmental cues that are required to reconstitute the tumor microenvironment [42]. Therefore, we confirmed our findings using a 3D culture system which at least partially recapitulates in vivo tumor growth conditions. We first determined LAPC4 spheroid growth following treatment with zol concentrations of 1, 3 and 10 µM in low serum conditions for 7 days. LAPC4 spheroid area was significantly decreased at 10 µM but not at lower concentrations (1 and 3 µM) after a week. Interestingly, the spheroids remained significantly smaller even when they were left up to 2 weeks in culture indicating continual action of zol on LAPC4 cell growth. The same observation was observed upon embedding LAPC4 with invasion matrix. In fact, LAPC4 spheroids were unable to invade the surrounding matrix but their surface area was significantly smaller at days 7 and 14 following treatment with zol at 10 µM. No effect of zol treatment at 1 or 3 µM was seen on spheroid growth and invasion of LAPC4. This could be expected in this type of 3D culture since most of the zol molecules, especially if low doses are tested, could be entrapped or slowly diffusing in the 3D microenvironment. The zol may therefore like other drugs be unable to optimally reach or penetrate the spheroids to exert their effects [43]. Additionally, it is well established that optimum growth conditions of tumor cells is different in 3D versus 2D cultures [42], and cells grown in 3D culture may be more resistant to drug treatment [43]. In this case, higher doses of drug may be required to manifest any effect on multiple cellular processes. When prostate-induced bone metastasis cells were treated with 10 µM zol for 1 week, both spheroid growth and invasion abilities were significantly reduced. Extending the incubation time for more than 7 days with 10 µM zol was toxic for prostate-induced bone metastasis spheroids both in the growth and invasion assays (data not shown). This could be explained by the fact that the prostate-induced bone metastasis cell spheroids could be more sensitive to treatment in 3D culture compared to spheroids from established and possibly heartier immortalized cell lines.

The current standard for delivering zol to patients is via intravenous infusion (single 4 mg dose for 15 min every 3–4 weeks). This systemic administration has multiple adverse effects, ranging from flu-like symptoms to osteonecrosis of the jaw and renal toxicity [44]. The systemic dose of zol to patients only maintains peak serum concentrations of 1–3 µM for few hours [45], as most zol has high affinity for mineralized bone [46]. Hence, the low doses of zol tested here, if adopted into clinical practice, would be insufficient to reach the tumor site and exert anti-tumor activity in a systemic manner. To circumvent these debilitating side effects and deliver the sustained low dose at the tumor site, efforts are being made to develop local delivery systems in preclinical studies. Interestingly, local zol treatment through either implant coating or topical application was proven efficient to enhance osteointegration or new bone formation [47]. We have also shown recently that local zol administration directly at the xenograft site via percutaneous catheter infusions inhibits tumor-induced osteolysis while revealing a trend for decreased tumor cell proliferation in a bone metastasis mouse model [36]. In the same study, local therapy of zol surprisingly outperformed systemic administration [36]. While this proof of concept is promising, the application of this method needs to be optimized since catheter administration of zol may not be the most practical clinical approach. Indeed, the use of an intradermal catheter to deliver chemotherapeutics/antiresorptives in cancer patients is not only subject to infections [48], but could also be incapacitating to patients during the treatment period. Progress has clearly been made in vitro and in vivo of material-based systems for local release of zol and/or other drugs such as the use zol-impregnated magnetic [49] or poly lactide-glycolide acid (PLGA) [50] nanoparticles, zol-loaded hydroxyapatite [51], calcium phosphate bone cement delivering zol [51–53] and nanohydroxyapatite/zol scaffolds [54]. Establishing a novel zol-loaded carrier as a new therapy strategy is therefore of important clinical significance since it would allow for effective and sustained local delivery of zol at the site of bone metastasis tumors following resection. These zol releasing systems would possibly inhibit tumor growth, enhance bone healing and limit the complications associated with systemic delivery.

To the best of our knowledge, this is the first study testing the anti-tumor effect of low-dose zol treatment on the prostate cancer cell line LAPC4 and primary cells from bone metastasis secondary to prostate. Some limitations however must be mentioned in this study. First, more primary cell cultures from patients with prostate-induced bone metastasis are needed to validate all the observations on a larger donor sample size. Second, the 3D in vitro model used to test invasion does not possess specific attributes of the bone system. Therefore, there is a need to establish a novel ex vivo bone model that recapitulates the bone metastasis microenvironment and provides a more physiological environment for study of tumor growth and invasion following anti-cancer drug treatment. Third, the described in vitro data requires validation in in vivo studies. Future work will apply this therapeutic approach to a xenograft bone metastasis animal model.

## Conclusion

In summary, we show that zol treatment in vitro at low concentrations reduces cell proliferation, migration and 3D growth/invasion of the prostate cancer cell line LAPC4 and the spine metastases cells secondary to prostate cancer. Additionally, the results shed new light on the use of low zol doses for longer duration as a new therapeutic regimen that will not only be sufficient to exhibit anti-tumor effects but could also prevent unwanted side effects following systemic drug treatment. These attributes could consequently lead to better quality of life and surgical outcomes in patients with bone metastasis.

## Additional file

**Additional file 1: Figure S1.** Cell proliferation using alamarblue® assay (a) and Vybrant MTT® assay (b) of LAPC4 cells treated with vehicle (PBS1x) or zoledronate 1 µM, 3µM, 10 µM and 100 µM for 14 days in 1% serum conditions. The histograms in (a) and (b) represent the ratio of drug-treated cells divided by vehicle-treated cells (PBS1x). (c) Histogram representing the ratio of live cells or dead cells in vehicle or zol-treated conditions of a Live/Dead assay, Results are from one experiment performed in triplicate, P < 0.05.

## Abbreviations

BPs: bisphosphonates
zol: zoledronate
SREs: skeletal related events
FBS: fetal bovine serum
PS: penicillin/streptomycin
EthD-1: ethidium homodimer-1
PLGA: poly lactide-glycolide acid
